# Impact of Age on Outcomes in Patients With Cardiogenic Shock

**DOI:** 10.3389/fcvm.2021.688098

**Published:** 2021-07-23

**Authors:** Manreet Kanwar, Katherine L. Thayer, Arthur Reshad Garan, Jaime Hernandez-Montfort, Evan Whitehead, Claudius Mahr, Shashank S. Sinha, Esther Vorovich, Neil M. Harwani, Elric Zweck, Jacob Abraham, Daniel Burkhoff, Navin K. Kapur

**Affiliations:** ^1^Cardiovascular Institute at Allegheny Health Network, Pittsburgh, PA, United States; ^2^The Cardiovascular Center, Tufts Medical Center, Boston, MA, United States; ^3^Beth Israel Deaconess Medical Center, Boston, MA, United States; ^4^Cleveland Clinic Florida, Weston, FL, United States; ^5^Massachusetts General Hospital, Boston, MA, United States; ^6^Department of Medicine, Division of Cardiology, University of Washington, Seattle, WA, United States; ^7^Inova Heart and Vascular Institute, Inova Fairfax Medical Campus, Falls Church, VA, United States; ^8^Northwestern Medicine, Chicago, IL, United States; ^9^Providence Center for Cardiovascular Analytics, Research, and Data Science, Portland, OR, United States; ^10^Cardiovascular Research Foundation, New York, NY, United States

**Keywords:** cardiogenic shock, age, mortality, mechanical circulatory support, outcome

## Abstract

**Background:** Advanced age is associated with poor outcomes in cardiovascular emergencies. We sought to determine the association of age, use of support devices and shock severity on mortality in cardiogenic shock (CS).

**Methods:** Characteristics and outcomes in CS patients included in the Cardiogenic Shock Work Group (CSWG) registry from 8 US sites between 2016 and 2019 were retrospectively reviewed. Patients were subdivided by age into quintiles and Society for Cardiovascular Angiography & Interventions (SCAI) shock severity.

**Results:** We reviewed 1,412 CS patients with a mean age of 59.9 ± 14.8 years, including 273 patients > 73 years of age. Older patients had significantly higher comorbidity burden including diabetes, hypertension and coronary artery disease. Veno-arterial extracorporeal membrane oxygenation was used in 332 (23%) patients, Impella in 410 (29%) and intra-aortic balloon pump in 770 (54%) patients. Overall in-hospital survival was 69%, which incrementally decreased with advancing age (*p* < 0.001). Higher age was associated with higher mortality across all SCAI stages (*p* = 0.003 for SCAI stage C; *p* < 0.001 for SCAI stage D; *p* = 0.005 for SCAI stage E), regardless of etiology (*p* < 0.001).

**Conclusion:** Increasing age is associated with higher in-hospital mortality in CS across all stages of shock severity. Hence, in addition to other comorbidities, increasing age should be prioritized during patient selection for device support in CS.

## Introduction

Cardiogenic shock (CS) is associated with high in-hospital mortality despite increasing use of temporary mechanical circulatory support devices (t-MCS) (1–3). Outcomes in CS depend on multiple factors including patient characteristics, hemo-metabolic profile and severity of CS on presentation. Although there is lack of high-quality randomized evidence to support their use in CS, t-MCS devices are increasingly available and patients previously considered too high-risk are now being supported with these devices (4, 5).

Age is a known, non-modifiable risk factor for mortality in patients with CS (6). Most of the published CS literature, including clinical trials has focused on shock resulting from acute myocardial infarction (AMI) (7–9). While the durable left ventricular assist device literature has extensively investigated outcomes in older patients, there remains a paucity of literature involving the use of t-MCS in this age-group (10, 11). The decision to place an older patient on t-MCS needs to consider their baseline functional status, comorbidities, physiological reserve and goals of care in a heightened fashion (12, 13). Since these are not well-studied, programs often choose somewhat arbitrary upper age limits for t-MCS use for CS patients at their sites (6).

With the introduction of the Society for Cardiovascular Angiography and Intervention (SCAI) CS stages, patients can now be classified consistently based on their severity of shock (14, 15). Recent reports have noted that older patients with CS have lower short-term survival, despite similar shock severity (16). We sought to describe the relationships between age, SCAI stage, use of temporary MCS and mortality risk in patients with CS included in the Cardiogenic Shock Work Group (CSWG).

## Methods

### Data Source

The CSWG is an academic research consortium with a national registry initiated in 2016 with 20 clinical sites across the United States contributing CS patient data. These sites include community and university hospitals with registry inclusion dependent on a minimum of 100 CS patients per year. For this analysis, CS patients at the first 8 sites contributing registry data between 2016 and 2019 were included. The registry includes a standardized set of data elements (patient, procedural, and outcomes) which were pre-defined by principal investigators and collected retrospectively. Patient demographic, laboratory and hemodynamic data were collected at a single time point as close to admission as possible, prior to t-MCS (i.e., intra-aortic balloon pump [IABP], Impella, veno-arterial extra corporeal membrane oxygenation [VA-ECMO], or extracorporeal centrifugal flow pumps) initiation. CS diagnosis was physician-adjudicated at each site and defined as a sustained episode of one out of the following: systolic blood pressure <90 mmHg for at least 30 min/use of vasoactive agents/a cardiac index (CI) <2.2 L/min/m^2^ in the absence of hypovolemia, determined to be secondary to cardiac dysfunction or use of an t-MCS device for clinically-suspected CS. Treatments for CS were left to the discretion of the clinicians at each center and were not guided by a prescribed algorithm. Quality assurance was achieved through adjudication at each site by the respective clinical coordinators and principal investigator. Values were centrally audited and screened by the CSWG research team for any discrepancies or major outliers and resolved with submitting site.

### Study Population

Between 2016 and 2019, data from 1,565 CS patients were collected. CS etiology was reported by each site as due to AMI, acute decompensated heart failure (ADHF), or other. AMI was defined as any primary diagnosis of either non-ST-segment elevation or ST-segment elevation AMI. ADHF was defined as any primary diagnosis of acute on chronic HF, not otherwise related to AMI. Other causes included post-cardiotomy, myocarditis, or not otherwise specified CS. We excluded patients under 18 years old (*n* = 1, 0.06%) and those with unknown in-hospital mortality status (*n* = 150, 9.6%) leaving a study population of 1,414 CS patients from 8 hospitals for analysis.

We then employed the recently published SCAI CS staging system to stratify this cohort by SCAI stage as we have previously described (15). SCAI Stage A patients are those at risk for CS and were therefore not captured in our study population. Stage B patients are those exhibiting early symptoms not including hypoperfusion and therefore do not require vasoactive medications or MCS. Stage C patients include those with hypotension and hypoperfusion requiring intervention beyond volume resuscitation including those requiring either one vasopressor/inotrope or one MCS device. Stage D patients are those whose condition deteriorates despite initial intervention, defined in our dataset by the need for multiple drugs or MCS devices. Finally, Stage E patients are those who deteriorate further and require maximal support, defined in our dataset as requiring at least two MCS devices and two drugs during their hospitalization. Patients requiring CPR on admission were included in Stage E.

### Statistical Analysis

Patients were divided into the following age quintile groups: age <49 years, 49–58, 59–65, 66–72, and >73 years. Quintiles were generated to ensure similar representation of number of patients for each decade of patient age. The primary outcome of interest was survival during index admission, determined using chart review. Continuous characteristics of each age cohort are displayed as means with standard deviations and *p*-values reported from ANOVAs. Categorical variables were expressed as frequency and percent and compared using chi-square tests of independence. Missing values were excluded where noted. To determine the impact of age on in-patient mortality, we ran a multivariable logistic regression adjusting for several potential confounders including gender, weight, history of hypertension (HTN), etiology of CS, systolic blood pressure, SCAI stage, renal function and cardiac power output. Results are reports as adjusted odds ratios with 95% confidence intervals. An alpha level of 0.05 was used to determine statistical significance throughout the entire analysis. All statistical analysis was performed using SAS 9.

## Results

### Study Population

Data from 1,412 CS patients from 8 clinical sites were analyzed. Baseline characteristics are summarized in Table 1. Of the study cohort, 1,025 (72.5%) patients were male and 493 (39.9%) presented with AMI-CS. The mean age of the combined cohort was 59.9 ± 14.8 years. The majority (*n* = 758, 53.6%) of patients were in SCAI stage D, with 263 (18.6%) in stage C and 212 (15%) in stage E shock (Table 2). CS was treated with vasoactive and/or pressor agents in 1,043 (73.8%) patients. MCS devices included IABP in 770 (54.5%), Impella® in 410 (29%) and VA-ECMO in 333 (23.6%) patients, with several patients receiving multiple devices (Table 3). Overall survival was 69.5% at the time of hospital discharge.

**Table 1:** Baseline characteristics for patients in cardiogenic shock, at the time of presentation, separated into quintiles by age.

	**All (*****N*** **=** **1,412)**		**Age quintiles**										
			**<** **49 (*****N*** **=** **284)**		**49–58 (*****N*** **=** **319)**		**59–65 (*****N*** **=** **268)**		**66–72 (*****N*** **=** **271)**		**73+ (*****N*** **=** **270)**		***p*-value**
	***N***	**(%)**	***N***	**(%)**	***N***	**(%)**	***N***	**(%)**	***N***	**(%)**	***N***	**(%)**	
Cause of shock													<0.001
Myocardial infarction	493	34.92	52	18.31	100	31.35	91	33.96	107	39.48	143	52.96	
Heart failure	712	50.42	165	58.1	168	52.66	152	56.72	133	49.08	94	34.81	
Other	177	12.54	61	21.48	43	13.48	22	8.21	25	9.23	26	9.63	
Unknown	30	2.12	6	2.11	8	2.51	3	1.12	6	2.21	7	2.59	
**Demographics**													
Male	1,025	72.59	201	70.77	247	77.43	204	76.12	193	71.22	180	66.67	0.03
Race													0.002
White	647	45.82	124	43.66	151	47.34	127	47.39	125	46.13	120	44.44	
Hispanic/Latino	31	2.2	8	2.82	11	3.45	3	1.12	4	1.48	5	1.85	
African-American	28	1.98	9	3.17	5	1.57	2	0.75	8	2.95	4	1.48	
Asian	31	2.2	6	2.11	5	1.57	7	2.61	6	2.21	7	2.59	
Unknown	593	42	103	36.27	133	41.69	113	42.16	117	43.17	127	47.04	
Medical history^*^													
Hypertension	681	53.54	79	30.27	117	41.2	143	57.66	154	64.17	188	78.66	<0.001
Diabetes	489	34.88	56	19.79	101	31.86	103	38.58	117	43.82	112	41.79	<0.001
A-fibrillation	296	29.16	36	15.65	62	27.31	74	36.27	68	36.36	56	33.53	<0.001
CKD	323	27.17	41	16.73	61	23.74	65	28.14	81	36	75	32.47	<0.001
PVD	60	5.82	1	0.47	5	2.21	14	7.07	15	7.69	25	12.5	<0.001
COPD	101	7.97	8	3.1	17	6.03	23	9.31	32	13.28	21	8.79	<0.001
CVA/TIA	159	12.92	22	8.73	22	8.06	33	13.58	39	16.96	43	18.45	<0.001
Valvular Ds.	214	22.55	39	18.93	45	21.33	41	21.58	44	24.72	45	27.44	0.34
Prior PCI	293	29.9	35	16.75	65	28.51	66	34.92	70	38.04	57	33.53	<0.001
Prior CABG	114	10.12	10	4.59	12	4.76	27	12.86	37	17.13	28	12.12	<0.001
VT	216	21.2	44	19.05	54	23.68	57	27.94	45	23.81	16	9.58	<0.001
ICD	329	32.8	79	34.65	90	40.36	75	37.31	65	34.95	20	12.12	<0.001
	**Mean**	**SD**	**Mean**	**SD**	**Mean**	**SD**	**Mean**	**SD**	**Mean**	**SD**	**Mean**	**SD**	
AST	459.41	1492.57	475.5	1504.46	518.83	1498.7	461.42	1793.04	581.01	1729.93	230.18	532.79	0.33
BUN	32.38	20.47	25.71	15.35	33.3	21.44	31.54	18.64	37.28	23.94	34.71	20.5	<0.001
Lactate	4.37	4.21	4.64	4.17	4.48	4.73	4.2	3.93	4.11	3.86	4.41	4.28	0.85
HCO_3_	22.12	5.45	22.92	5.53	22.06	5.77	22.1	5.63	22.06	5.1	21.45	5.08	0.18
Serum creatinine	1.76	1.14	1.61	1.26	1.69	0.96	1.76	1.11	1.97	1.25	1.8	1.09	<0.001
pH	7.31	0.15	7.3	0.17	7.29	0.14	7.3	0.15	7.32	0.15	7.33	0.13	0.28
Admission EF (%)	24.94	15.53	22.73	16.48	21.5	13.57	21.54	12.93	25.19	14.83	32.6	16.79	<0.001
RAP	14.19	6.93	13.49	6.54	14.63	7.32	14.24	7.32	14.27	7.27	14.35	5.95	0.49
PCWP	24.5	8.9	23.7	9.03	24.31	8.55	25.13	9.36	24.67	9.13	24.87	8.41	0.61
Mean PAP	32.73	9.86	32.66	10.21	32.48	9.7	32.85	10.21	33.21	10.01	32.51	9.12	0.94
CPO	0.63	0.41	0.69	0.57	0.65	0.36	0.62	0.44	0.63	0.36	0.55	0.21	0.02
Heart rate	92.02	22.72	99.11	25.54	93.05	20.64	91.79	21.7	89.74	21.67	85.42	21.7	<0.001
Cardiac index	1.85	0.59	1.89	0.66	1.82	0.53	1.84	0.56	1.86	0.6	1.84	0.61	0.72
MAP	74.56	14.75	74.7	15.33	75.43	14.94	73.71	13.31	73.95	14.99	74.82	15.06	0.66
SBP	98.17	20.02	95.39	18.27	96.22	18.39	97.99	18.92	98.91	21.89	102.99	21.93	<0.001
GFR	48.86	21.38	57.07	21.69	50.26	20.7	47.64	20.18	42.13	21.45	44.95	19.57	<0.001

* *Percentages and chi square tests of independence do not include missing values*.

*CKD, chronic kidney disease; PVD, peripheral vascular disease; COPD, chronic obstructive pulmonary disease; CVA, cardiovascular accident; TIA, transient ischemic attack; PCI, percutaneous intervention; CABG, coronary artery bypass graft; VT, ventricular tachycardia; ICD, implantable cardioversion-defibrillator; SD, standard deviation; AST, aspartate transaminase; BUN, blood urea nitrogen; EF, ejection fraction; RAP, right atrial pressure; PCWP, pulmonary capillary wedge pressure; PAP, pulmonary artery pressure; CPO, cardiac power output; MAP, mean arterial pressure; SBP, systolic blood pressure; GFR, glomerular infiltration rate; SCAI, Society of Cardiovascular Angiography and Intervention; IABP, intra-aortic balloon pump; VA-ECMO, veno-arterial extra-corporeal membrane oxygenator*.

**Table 2:** Distribution of SCAI stages across age quintiles.

**SCAI stage**	**All (*****N*** **=** **1,412)**		**Age quintiles**										***p*-value**
			**Age** **<** **49 (*****n*** **=** **284)**		**49–58 (*****n*** **=** **319)**		**59–65 (*****n*** **=** **268)**		**66–72 (*****n*** **=** **271)**		**>73 (*****n*** **=** **270)**		
	***N***	**%**	***N***	**%**	***N***	**%**	***N***	**%**	***N***	**%**	***N***	**%**	
B	46	3.26	16	5.63	9	2.82	9	3.36	6	2.21	6	2.22	0.005
C	263	18.63	53	18.66	69	21.63	36	13.43	47	17.34	58	21.48	
D	758	53.68	146	51.41	150	47.02	152	56.72	156	57.56	154	57.04	
E	212	15.01	45	15.85	62	19.44	48	17.91	34	12.55	23	8.52	
Unknown	133	9.42	24	8.45	29	9.09	23	8.58	28	10.33	29	10.74	

**Table 3:** Device distribution across age quintiles.

**Treatment**	**All (*****N*** **=** **1,412)**		**Age quintiles**										***p*-value**
			**Age** **<** **49 (*****n*** **=** **284)**		**49–58 (*****n*** **=** **319)**		**59–65 (*****n*** **=** **268)**		**66–72 (*****n*** **=** **271)**		**>73 (*****n*** **=** **270)**		
	***N***	**%**	***N***	**%**	***N***	**%**	***N***	**%**	***N***	**%**	***N***	**%**	
# Devices													<0.001
0	223	15.79	69	24.3	52	16.3	39	14.55	30	11.07	33	12.22	
1	881	62.39	148	52.11	184	57.68	163	60.82	184	67.9	202	74.81	
2	271	19.19	60	21.13	71	22.26	56	20.9	53	19.56	31	11.48	
3	36	2.55	7	2.46	12	3.76	9	3.36	4	1.48	4	1.48	
4	1	0.07	0	0	0	0	1	0.37	0	0	0	0	
**Device type**													
VA-ECMO	332	23.51	101	35.56	91	28.53	63	23.51	52	19.19	25	9.26	<0.001
Impella	410	29.04	62	21.83	93	29.15	94	35.07	83	30.63	78	28.89	0.02
IABP	770	54.53	122	42.96	176	55.17	140	52.24	162	59.78	170	62.96	<0.001
Mechanical ventilation^*^	571	58.62	123	55.66	134	59.56	118	60.51	101	56.74	95	61.29	0.76
Medical therapy^*^	1,043	81.55	216	83.08	237	81.72	213	86.94	197	81.07	180	74.69	0.01

* *Percentages and chi square tests of independence do not include missing values*.

### Patient Characteristics Across Age Groups

The distribution of patients across the age quintiles is displayed in Table 1. Older patients (age > 73) were more likely to be female and present with AMI as their etiology for CS compared to their younger counterparts. Patients above 66 years of age had a higher comorbidity burden, with a higher likelihood of Type 2 Diabetes (DM2) and prior percutaneous coronary intervention (*p* < 0.001). The prevalence of HTN and stroke increased with each quintile (*p* < 0.001). Prior to device implantation, all patients had comparable lactate and bicarbonate levels but older patients (66 and older) had significantly higher serum creatinine (*p* < 0.001) compared to younger patients. Filling pressures prior to device implantation were also comparable across all age groups; however, older patients were more likely to have right sided congestion. The distribution of SCAI shock stages differed across age groups, with a higher prevalence of SCAI shock stage C/D in older patients (66 and older).

### Analysis of Mortality During Index Admission

**Figure 1:**
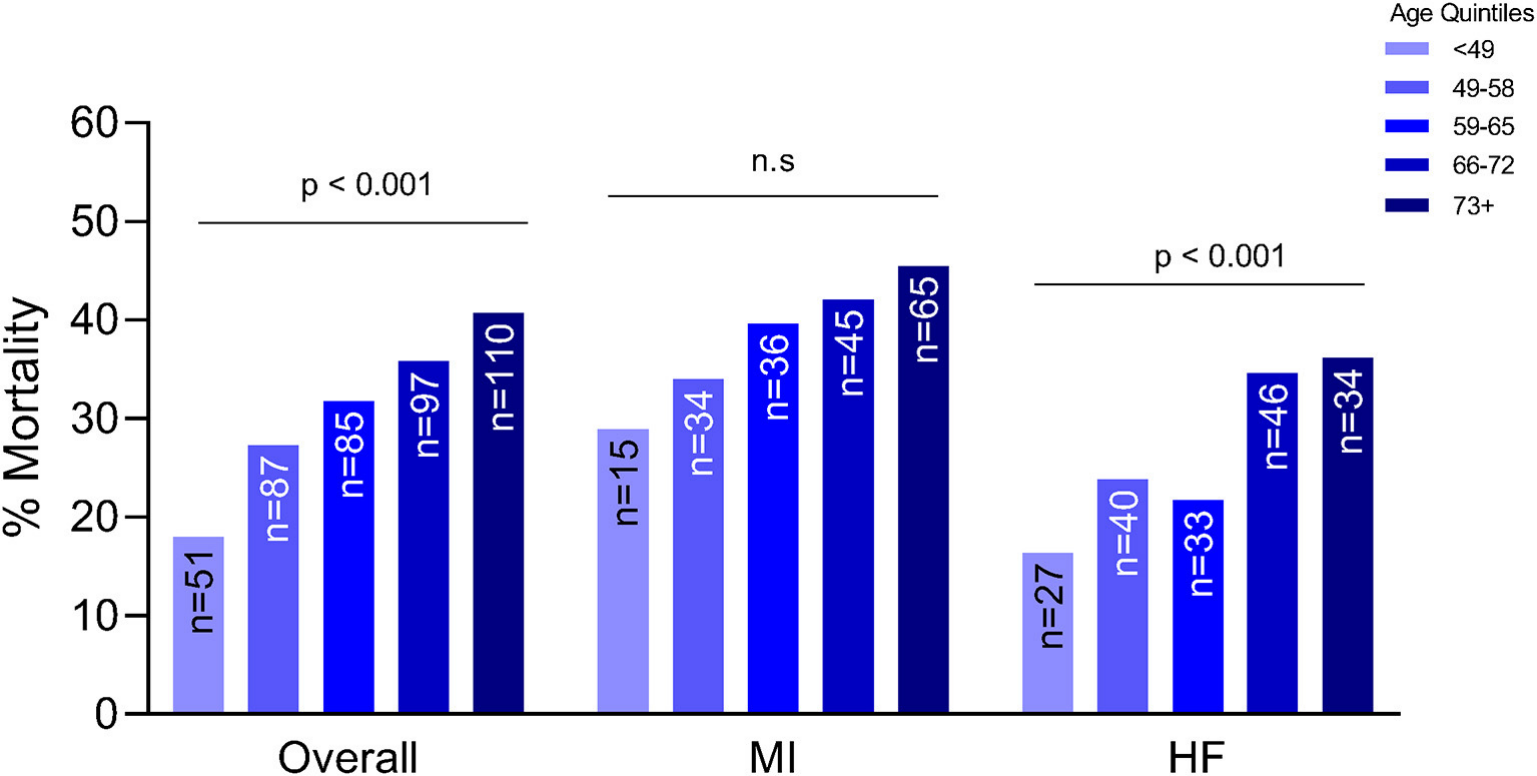
Association between age and mortality by etiology of cardiogenic shock. MI, myocardial infarction; HF, heart failure; n.s., not significant statistically.

**Figure 2:**
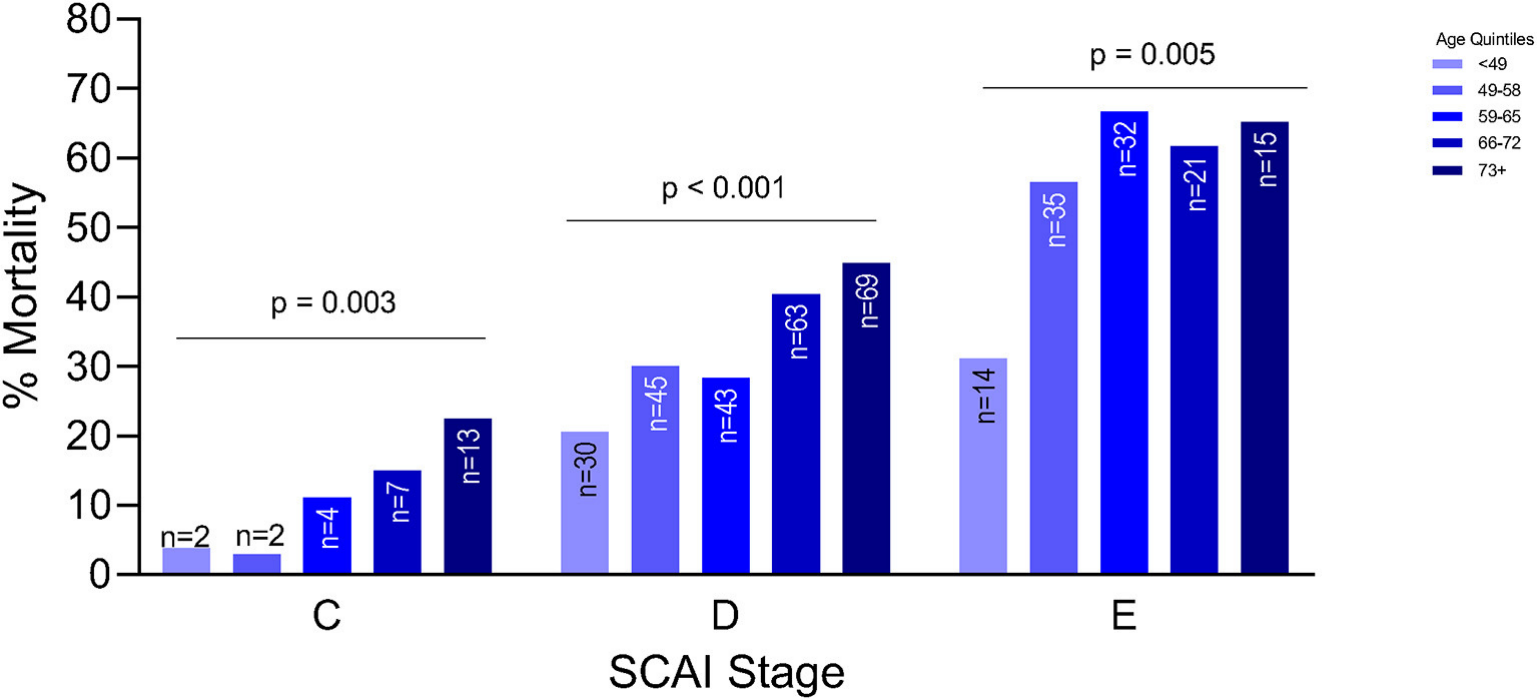
Association between age and mortality by severity of cardiogenic shock as defined by the Society of Cardiovascular Angiography and Intervention (SCAI) classification.

Older patients were at a higher risk of mortality, regardless of etiology (*p* < 0.001) (Figure 1). Although this trend was seen in both etiologies, the trend was statistically significant in patients with ADHF (*p* < 0.001) compared to the MI group. After adjusting for gender, weight, history of HTN, etiology, systolic blood pressure, SCAI stage, renal function and cardiac power output, each increase in age by quintile was significantly associated with 1.47 times the odds of in-hospital mortality (OR: 1.47, 95% CI: 1.20–1.79). Worsening SCAI stages were associated with a higher risk of mortality and within each stage, there was a higher risk of mortality with increasing age (*p* = 0.003 for SCAI stage C; *p* < 0.001 for SCAI stage D; *p* = 0.005 for SCAI stage E) (Figure 2).

### Use of t-MCS Across Age Group

**Figure 3:**
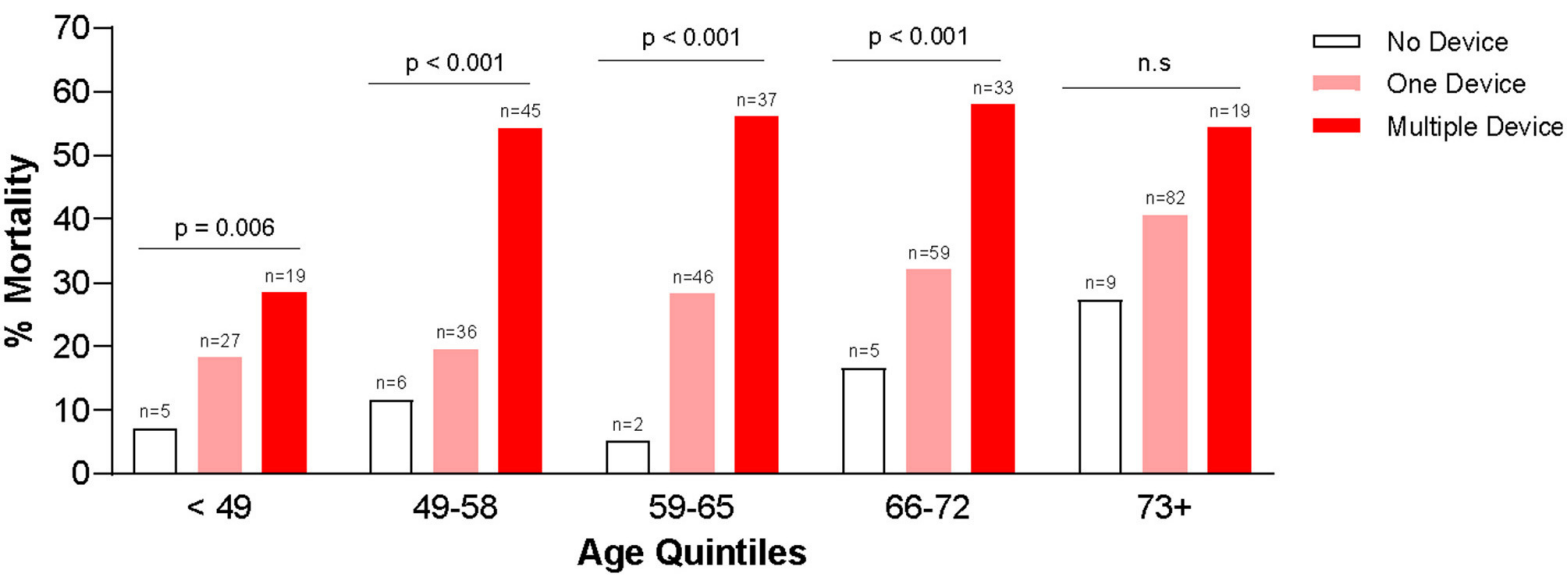
Association between in-hospital mortality and use of temporary mechanical circulatory support devices.

Table 3 summarizes the use of t-MCS devices in each quintile of age groups. Several (*n* = 99, 7.0%) patients received multiple MCS devices during their hospitalization, especially in the first, second and third quintile. In most age groups, getting multiple devices was associated with worse outcomes. In fact, risk of mortality was higher with increasing age, regardless of whether the patient was supported on any t-MCS device(s) or not (Figure 3).

## Discussion

We describe the association between age, severity of CS and use of t-MCS devices in one of the largest multi-center registries representing real-world CS patients in the contemporary era. Older age was associated with higher mortality that was additive to the effect of shock severity. Higher SCAI shock stages were associated with increased mortality risk in each age group, while older patients were more likely to die at each level of shock severity. The use of t-MCS was consistently associated with a higher mortality across each age group, regardless of severity of CS. This study provides real-world survival estimates for CS patients as a function of both age and shock severity.

Age has been identified as a major risk factor for both short and long-term mortality in patients with CS. Age cut-offs ranging from 60 to 75 have been proposed as thresholds for prediction of higher mortality in CS. Similar age cut-offs have been suggested for use of ECMO as therapy for CS, although its use in older patients remains controversial (6, 17). Although these studies have highlighted the impact of age on outcomes in CS, they have not accounted for the severity of CS. Moreover, the majority of published analyses have focused on CS from AMI. A recent 2 center study reported congruent findings of graded relationship between older age and lower survival in CS that was additive to the level of shock severity (16). Although CS was identified using a diagnosis code and a large percentage of patients were in early, stage B shock, our findings strengthen their observation that age and increasing shock severity are associated with worse outcomes. Our study expands on these prior analyses by including both AMI-CS and HF-CS patients, further stratified by the severity of CS using the SCAI shock stages and including a large number of patients in advanced stages of shock.

Numerous age-related factors can potentially contribute to worse outcomes in older patients, including frailty and reduced functional reserve, delayed or atypical clinical presentation as well as multiple comorbidities. Not surprisingly, older patients were more likely to have DM2 and hypertension, and more likely to have undergone prior percutaneous coronary intervention in our cohort. In our analysis, increasing age continued to be associated with higher odds of mortality after adjusting for the known risk factors such as gender, SCAI stage, renal function, cardiac power output etc. Recent data suggests that survival of CS patients > 65 years requiring ECMO is poor and less commonly includes transition to definitive advances therapies (18). Our data further suggests that age modifies the relationship between severity of shock and mortality in CS patients, especially considering that the hemodynamic and metabolic profiles are so evenly distributed across the age groups. These comorbidities become especially relevant in establishing goals of care for the older population.

For some other cardiovascular diseases such as aortic stenosis, older individuals who undergo trans-catheter aortic valve replacement (TAVR) are now experiencing comparable in-hospital recovery, and similar short and mid-term mortality compared to their younger counterparts (19). Similarly, revascularization has been shown to improve mortality in older patients with AMI complicated by CS in some reports but not in others (20–22). Although these reports are encouraging for management of common cardiovascular comorbidities such as CAD and aortic stenosis in the elderly, it is not enough reason to believe that this improvement in outcomes will be extended to a high risk scenario or aggressive interventions such as ECMO support in CS. CS is a very complicated illness to manage, often requiring significant time in intensive care, undergoing invasive therapies. Advancements in t-MCS technology have made this therapeutic modality more widely available; yet, they are associated with various inherent risks, including vascular complications, risk of infection and bleeding (23). Older adults with decreased physiologic reserve may be less likely to withstand such complications in order to derive the benefits provided by this therapy. This should be especially taken into consideration while managing older patients with CS since they may or may not be in favor of aggressive and invasive therapies in the setting of critical illness.

Selection of therapies, especially t-MCS in CS patients is never straightforward and has to be individualized based on baseline characteristics, etiology, clinical presentation and goals of care. While biological age should be used as one of multiple clinically relevant factors in the decision-making process, it is important to remember that older patients may have different goals of care than younger patients. However, numerical age by itself should not preclude patients from t-MCS. Especially in cases of AMI, patients are often critically ill when they arrive at the hospital, and clinicians have insufficient time and clinical information about the patient's risk factors to make well-informed decisions. Our data reveal the marked rise in risk of mortality with use of t-MCS for older patients, regardless of severity of shock. This information can be reasonably be used to help providers determine best approach to an individual patient and inform patients and families about expected outcomes with a clearer explanation of risks and benefits. In the second iteration of the CSWG registry, participating sites are now collecting data on not just survival but adverse events, including vascular complications that result from a combination of CS and therapeutic interventions. This is essential, since quality of life and risk of AEs are often equally important as survival, especially in the elderly.

Our data are retrospective in nature and come with inherent limitations. Several confounding variables (e.g., frailty, nutritional status, baseline functional assessment, goals of care) remain unmeasured. Decisions to proceed with t-MCS (or not) were made by individual treating physicians, introducing a selection bias which may favor higher use of devices in younger patients. We are not aware of the “code-status” of included patients which would also direct treatment strategies. We did not collect the timing of device therapies relative to each other in those who received multiple devices. However, our real-world, multi-center registry report of more than 1,400 CS patients helps highlight the additive impact of age on shock severity when risk-stratifying these patients. Our ongoing data collection will allow us much more in-depth analysis of patient's hospital course, and will allow us to suggest an age “cut-off” for different scenarios in CS to try and answer the question “how old is too old” for t-MCS. More importantly, our future analyses may allow us to identify characteristics in the older patients that promote survival benefit with t-MCS in-spite of advanced age (e.g., reversible etiology of CS, post-cardiotomy, time to ECMO etc.). Lastly, acknowledging that survival at discharge is not the only goal with t-MCS, we are now collecting 30 day and 1-year outcomes in all patients which will add significant value to this discussion.

## Conclusions

Increasing age is associated with a higher mortality in CS, regardless of shock severity. Use of t-MCS devices is associated with increased mortality in all age groups and SCAI stages. Given the poor outcomes observed in the older patients, identifying selected patients who may benefit from more aggressive treatment strategies despite advanced age is a major unmet need. This would allow for a more informed risk stratification strategy in this critically ill patient population.

## Data Availability Statement

The original contributions presented in the study are included in the article/supplementary material, further inquiries can be directed to the corresponding author/s.

## Author Contributions

MK, AG, SS, DB, and NK contributed to concept, data analysis, writing, and review. KT and NH for data analysis. JH-M, EW, CM, EV, EZ, and JA to data contribution, writing, and review. All authors contributed to the development and writing of the manuscript.

## Conflict of Interest

MK and JH-M are consultants for Abiomed Inc. CM is a consultant for Abbott Laboratories, Abiomed Inc., Medtronic, and Syncardia. AG is a consultant for Abiomed and NuPulseCV. He has received research support from Abbott and Verantos. DB reports an unrestricted, educational grant from Abiomed Inc. to Cardiovascular Research Foundation. JA was a consultant for Abbott Laboratories, Abiomed Inc. JH-M receives speaker honoraria and a research grant from Abiomed Inc. NK receives consulting/speaker honoraria and institutional grant support from: Abbott Laboratories, Abiomed Inc., Boston Scientific, Medtronic, LivaNova, MDStart, and Precardia. The remaining authors declare that the research was conducted in the absence of any commercial or financial relationships that could be construed as a potential conflict of interest.

## Publisher's Note

All claims expressed in this article are solely those of the authors and do not necessarily represent those of their affiliated organizations, or those of the publisher, the editors and the reviewers. Any product that may be evaluated in this article, or claim that may be made by its manufacturer, is not guaranteed or endorsed by the publisher.

## Abbreviations

t-MCS: temporary mechanical circulatory support
CS: cardiogenic shock
SCAI: Society for Cardiovascular Angiography & Interventions,
MI: myocardial infarction
VA-EMCO: Veno-arterial extracorporeal membrane oxygenation
IABP: intra-aortic balloon pump
HF: heart failure.
